# Thoracic endovascular aortic repair in penetrating aortic ulcer combined with isolated left vertebral artery: A case report

**DOI:** 10.1097/MD.0000000000017159

**Published:** 2019-09-13

**Authors:** Weijian Fan, Chuanyong Li, Guangfeng Zheng, Zhichang Pan, Jianjie Rong

**Affiliations:** Department of Vascular Surgery, Suzhou TCM Hospital Affiliated to Nanjing University of Chinese Medicine, Suzhou, Jiangsu Province, China.

**Keywords:** chimney technique, in situ fenestration, isolated left vertebral artery, penetrating aorta ulcer, thoracic endovascular aortic repair

## Abstract

**Rationale:**

Penetrating aorta ulcer (PAU) with isolated left vertebral artery (ILVA) is a rare condition, accounting for no more than 1% of all kinds of aorta diseases. And traditional treatment was open surgery with total arch replacement by elephant trunk. Here, we report a case of PAU combined with ILVA managed by thoracic endovascular aortic repair (TEVAR) technique.

**Patient concerns:**

A 65-year-old male with chronic hypertension and Nicotine abuse underwent intermittent back pain for 2 years and aggravated a bit for 1 week.

**Diagnoses:**

Preoperative computed tomography angiogram (CTA) indicated PAU combined with ILVA.

**Interventions:**

TEVAR was performed for PAU following with retrograde in situ fenestration and chimney technique for revascularization of ILVA and left subclavian artery (LSA), respectively.

**Outcomes:**

The operation was successfully and the patient was discharged from hospital after 1 week of treatment. Postoperatively, the images of CTA illustrated the patency of aorta, ILVA, and LSA without obvious endoleak. Besides, no ischemia attack or other relative syndromes were detected at 6-months follow-up.

**Lessons:**

This case demonstrates that TEVAR is an alternative to elephant trunk especially for PAU with ILVA. And it also showed the precise exposure of ILVA and necessity to reconstruct ILVA during TEVAR operation in order to reduce the occurrence of ischemia attack.

## 1 Introduction

Aorta rising from left ventricular anatomically is predominant among the process of circulation and metabolism. One primary problem is that aortic arch diseases, including acute aortic dissection, aneurysm, penetrating aortic ulcer, and even thoracic aorta coarctation overwhelmingly result in unfavorable clinical outcomes and complicated surgical challenges.^[1]^ Recent theoretical developments have revealed that severe complications (cerebral ischemia and stroke, visceral, renal, and limb ischemia) relating to conventional replacement of aortic arch lead to high morbidity and mortality rates while thoracic endovascular aortic repair (TEVAR) is associated with better outcomes especially in some conditions when lesions are located restrictively away from ascending aorta.^[2]^ A challenging problem which arises in this domain is the reconstruction of 3 branches on aortic arch and this appears as a more straightforward problem when facing with the isolated left vertebral artery (ILVA), arising directly from aorta arch instead of left subclavian artery (LSA), presenting as one of the most common aortic arch abnormalities. This turns out to be even more problematic because it is rare and is likely to increase the risk for cerebral ischemia during revascularization when aortic lesions are combined with ILVA. This report demonstrates a successful TEVAR operation in treatment of penetrating aortic ulcer combined with ILVA.

## 2 Case presentation

Approval was obtained from the Ethics Committee of the Affiliated Suzhou Chinese Traditional Medicine Hospital, Nanjing University of Chinese Medicine for presenting this case.

A 65-year-old Chinese male with intermittent back pain for nearly 2 years and aggravated a bit this week. He underwent an emergent enhanced chest computed tomographic scan to reconstruct the morphology of aorta arch and cervical arteries, which leading to a systematic diagnosis of penetrating aortic ulcer (PAU). He had a long history of hypertension and smoking while the vital signs were normal as well as physical examination. In terms of other examinations, the laboratory test illustrated that the account of leukocyte was 5.71 × 10^9^ cells/L, the level of C-Reactive Protein (CRP) was 3.50 mg/dl while the volume of low density lipoprotein (LDL) was 4.55 mmol/L As well as triacylglycerol (TC) was 2.71 mmol/L, which turned to reveal that the patient positively had a long history of hyperlipemia.

**Figure 1:**
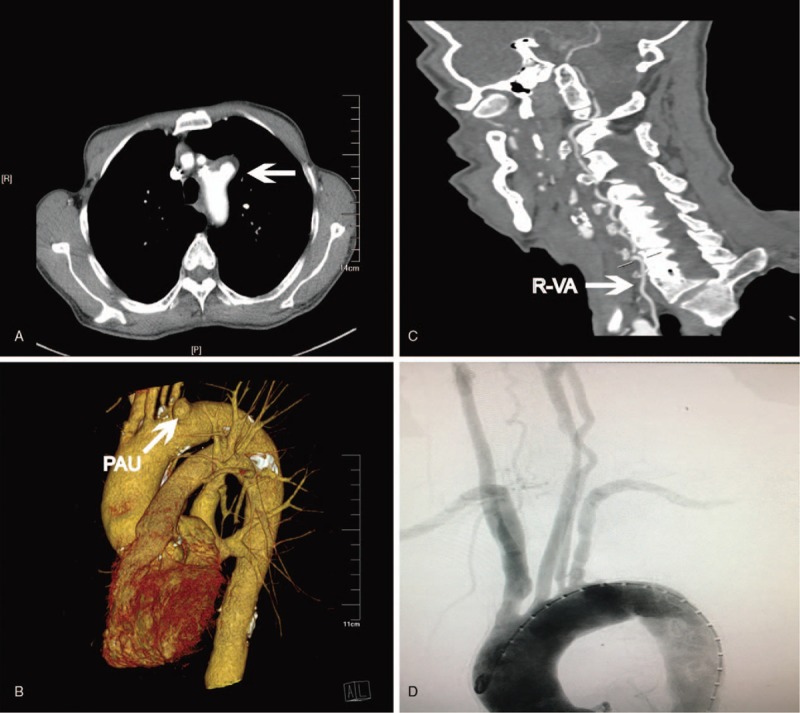
Preoperative enhanced CTA (A, B) showed penetrating aortic ulcer and calcific plaque of aorta arch (White arrow). Enhanced CTA (C) illustrated R-VA was found tenuous and insufficient (White arrow). Digital subtraction angiography (D) revealed type II arch and ILVA arising from the aorta adjacent to PAU.

According to the computed tomography angiography (CTA), the penetrating aortic ulcer was totally confirmed as well as extensive calcific plaque on aorta arch. The PAU appeared like cylinder and was located at the anterior wall of aorta arch with a maximal diameter of 14 mm and was only 11 mm adjacent to left common carotid artery (LCCA). Besides that, ILVA was depicted arising from the arch wall and close to the PAU. (Fig. 1) Due to the confirmation of diagnosis, consistent syndromes and patient's desire, TEVAR was prepared to revascularize the aorta arch combining with reconstruction of branches including chimney technique and in situ fenestration.

**Figure 2:**
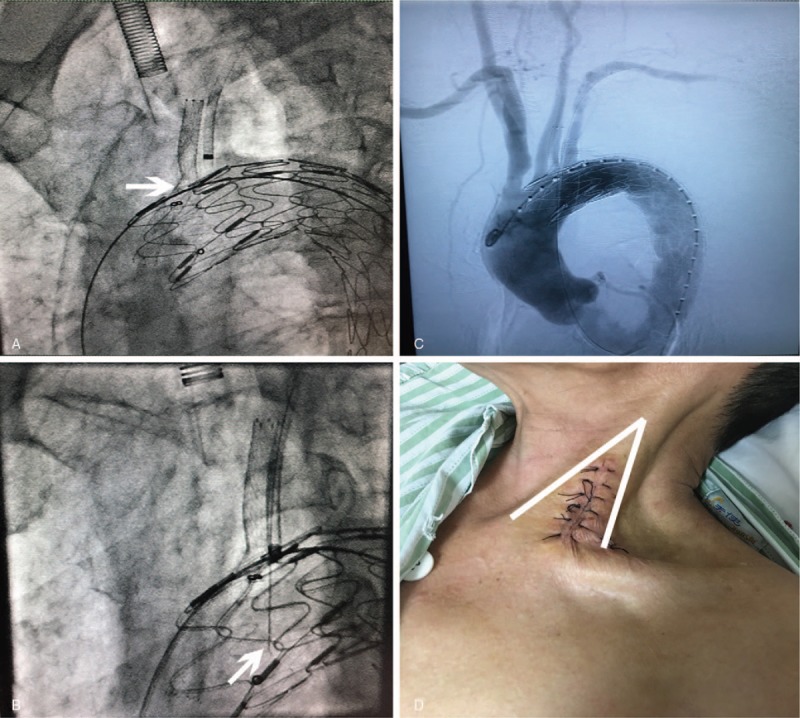
Perioperative angiography (A) demonstrated that the graft was deployed and chimney stent was implanted through the sheath in ILVA (White arrow). Perioperative fluoroscopy (B) presented that the needle was punctured through the graft into the true lumen of aorta. Eventual angiography (C) revealed the reconstruction of branches after covered graft was deployed, an eradication of PAU and the patency of ILVA as well as LSA. Post-operative picture (D) showed the anatomical situation on neck for exposure of ILVA.

The patient was routinely positioned supine and treated with general anesthesia. Due to the images of CTA, TEVAR technique was intended to proceed with reconstruction of ILVA and LSA. Both right femoral artery and left brachial artery were surgically exposed, punctured using Selindler technique and implanted with 12-F short sheath and 8-F short sheath, respectively. For exposure of ILVA, the incision was located at the interval of sternocleidomastoid branches, one attaching on manubrium while the other attaching on proximal part of the clavicle, in accordance with their relationships from chest and cervical CT and then, an 8-F sheath was introduced into ILVA. Through preserved sheath in LSA, super smooth guidewire combined with a marked pigtail catheter with scales was advanced into ascending aorta and an initial angiography was performed for further confirmation of preoperational diagnosis, PAU. After measurements of aorta arch and descending aorta from angiogram, 0.035-inch super stiff guidewire (Amplatz, Olympus, USA) was introduced via right femoral artery access, advanced across the PAU softly, ended at ascending aorta. Immediately following that, a 36 × 200 mm covered graft (Ankura, lifetech, China) which was chosen under intra-operative assessment was transported through guidewire and positioned at the right side of innominate artery. Meanwhile, another 2 super stiff guidewires were interpositioned into ascending aorta both through vertebral and brachial artery sheaths. The systolic blood pressure was significantly controlled to 110 mmHg during the implantation of endograft. The whole system was withdrawn a little, and completely released when the bare metal part of graft was anchored at the right side of LCCA and the proximal edge of covered part was positioned at the right side of ILVA. After that the systolic BP was elevated to 160 mmHg and a covered stent (viabhan, Gore, USA) was advanced through the guidewire in ILVA sheath to revascularize ILVA by utilizing “Chimney technique”. Following that, another self-expanded bare stent was transported and released inside the former one and 4 × 20 mm balloon was exchanged to dilate the channel. Furthermore, an 8-F guiding catheter was advanced through the preserved guidewire from left brachial access and interpositioned at the origin segment of LSA. The 0.035-inch stiff guidewire was withdrawn through the catheter and exchanged with 0.018-inch guidewire, which the distal stiff end was handmade into sharp needle shape preoperatively. The guiding catheter was controlled stably to the fiber surface of graft and needle was manipulated to create in situ fenestration after multiple endeavors. Fluoroscopy was performed in different dimensions for ensuring about successful breaking through and the guidewire was situated into the true lumen of aorta. After confirming the reverse blood flow, an 8 × 37 mm and 10 × 40 mm balloon was introduced across the hole to enlarge the fenestration through this unique wire. Furthermore, across the fenestration, an 8 × 10 mm covered stent as well as 7 × 20 mm self-expanded stent were inserted to reconstruct the LSA and followed with post-dilation by 8 × 20 mm balloon. Afterwards, angiography was performed and demonstrated a precise location of endograft, and exclusion of PAU and patent ILVA and LSA. Besides, the aortography illustrated the patency of LCCA and innominate artery while no obvious endoleak was found in sight. (Fig. 2) The graft transportation system and all guidewires were withdrawn as well as incisions were closed carefully. Intravenous heparin 3000 U and 30 U/min drip infusion of heparin were administrated and antibiotics therapy was proceeded peri-operationally.

**Figure 3:**
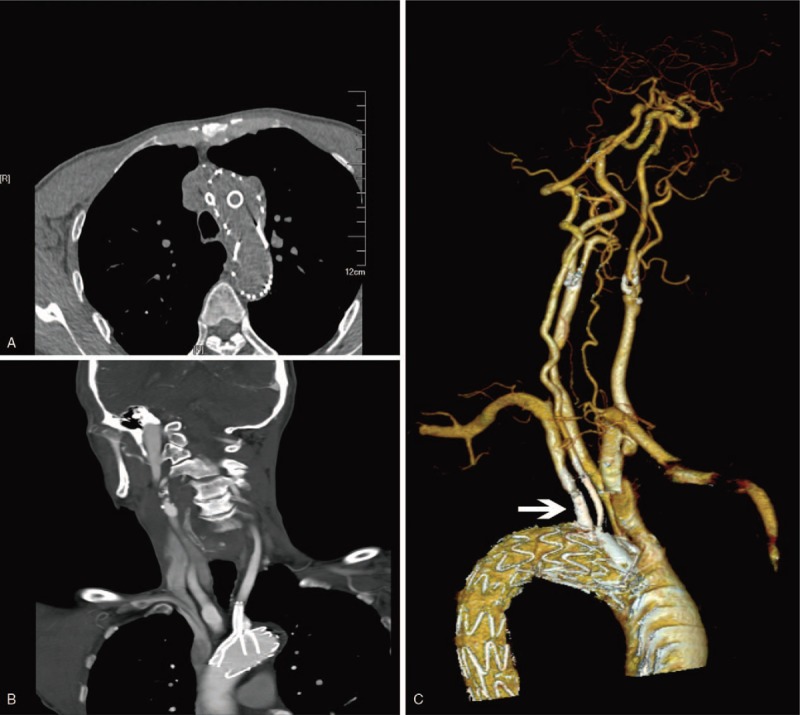
After 6 months follow-up, enhanced CTA (A and B) showed the significant primary patency of ILVA and LSA. CTA (C) illustrated the favorable revascularization of aorta arch and no evidence of endoleak.

The operation proceeded well and the patient had no symptoms of stroke or spinal cord ischemia following the procedure. At 6-month follow-up with CTA, the patient was presented with the patency of main graft, ILVA and LSA without any in-stent stenosis and no evidence of endoleak. (Fig. 3) No transient ischemia attack and stroke was found as well as no further episodes of back pain.

## 3 Discussion and conclusions

Total arch replacement and distal anastomosis were initially utilized in treating with aortic arch diseases, which contains some paramount techniques, like cerebral protection, managements of the nasopharyngeal temperature and establishments of circulatory arrest. Fortunately, The elephant trunk technique was first introduced in 1983 and hitherto, brought new remedy as well as a high risk of post-operative mortality and neurological events for mid-term follow-up.^[3]^ Furthermore, TEVAR which represents as second-stage of treatments in aortic arch diseases has showed its advantages of markedly shortening the procedural time, minimally invasive nature and no need for construction of carotid-subclavian bypass.^[4]^ Recent reports illustrate an in-hospital mortality of between 4.5% and 11.1%, a pooled mortality of 6.1% and spinal cord ischemia of 4.6% during mid-term follow up.^[5]^

In terms of TEVAR, multitude methods have been widely used in revascularization of arch branches, the vital step during the whole process, including chimney, in situ fenestration and customized endograft. However, customized graft is off-label in China, so that techniques including handmade needle,^[6,7]^ radiofrequency,^[8,9]^ and laser associated with dilation balloon (conventional or cutting)^[10,11]^ were overwhelmingly chosen for in situ fenestration.

Pre-operational CTA showed that the right vertebral artery (R-VA) was congenital tenuous and distorted while the distal segment of it ended for ipsilateral posterior inferior cerebellar artery (PICA) solitarily. Besides, the angiography illuminated that ILVA was predominant compared with R-VA for posterior circulation. Research on Willis circle has a long tradition and the prevalence of a complete circle of Willis was 42% in a general Western population as reported by Krabbe-Hartkamp and colleagues.^[12]^ However, a complete circle of Willis was seen in only 27% of Chinese people in a recent report.^[13]^ In this way, there has been less previous evidences whether the ILVA played a leading role in the main blood supply to the brainstem and cerebellum or whether the arterial communication at the circle of Willis was inadequate.

This field closely follows the paradigm that a type III of aortic arch is highly recommended to be implanted with a chimney graft which can reduce the time range in dealing with aortic arch type III and reconstructing the carotid artery compared with in situ fenestration.^[10]^ For this study, it was of interest to deploy a chimney graft in order to revascularize the ILVA. Owing to the significance of keeping the patency of ILVA, chimney technique was more prompt for reconstruction of ILVA. An apparent difficulty of the case was the exposure of ILVA, where the anatomy of ILVA is far more disparate from normal structure, the first branch of LSA, arising through the transverse foramen of cervical vertebra. However, in terms of ILVA, the origin segment was started from aorta while the V2 segment entered through cervical vertebra regularly which means the dissociative part of ILVA was shorten for sheath insertion. The precise incision site for the exposure of ILVA was shown between the intervals of sternocleidomastoid branches as described before in Figure 2.

Nevertheless, the process of in situ fenestration was performed while the cerebral blood flow (CBF) was blocked temporarily. By doing so, multiple attempts of in situ fenestration would prolong the revascularizing time of ILVA, lead to the deficiency of CBF and induce transient ischemia attack (TIA) or minor stroke of post cerebral circulation potentially when combing with contralateral arterial lesions, even if recent researches demonstrated that CBF can be safely interrupted for 5 minutes at room temperature.^[14,15]^ Therefore, revascularization of ILVA would bring benefits to cerebral circulation and reduce the ischemia risk both in cerebellum.

In terms of technique, the approach utilized suffers from the limitation that chimney graft would possibly enlarge the risk of endoleak.^[16,17]^ Therefore, the present findings confirmed that the PAU was located at the anterior wall of aorta arch, so that the graft we chose was oversized, nearly 10% to 15% larger than the precise diameter of aorta and the chimney stent was implanted at the posterior wall of graft in order to decrease the risk of endoleak.

The previous study about laser fenestration demonstrated uncertainty that the PTFE and Dacron fabrics of graft would easily be tore and carbonated so that the generating tears and other substances probably pose unknown influences to metabolism of both brain and other peripheral systems.^[18,19]^ Also, cutting balloons increases fabric fraying and tears in stent grafts.^[20]^ Moreover, some articles maintain that both vessel walls of aorta would be possibly perforated by needle fenestration.^[18]^ However, in our center, a number of works have shown that injury to aorta arch can be overcome by experienced doctors because the diameter of aorta is wide enough and the distance between brachial incision and aorta arch is close, which means the guide wire and catheter are easily under controlled.

No study to date has examined the optimal choice of graft during TEVAR, to our knowledge, Relay graft, Medtronic graft and Ankura graft were widely utilized. However, regarding of the obvious limitations of the soft delivery sheath in supporting, it could be manifested that the Relay thoracic stent graft is normally considered unsuitable for implementation in certain settings.^[21]^ Furthermore, in consideration of needle technique, it is important to highlight the fact that the Ankura graft, thanks to its thinner fabric intima than Medtronic graft, turned to be more feasible for in situ fenestration. Also, it is by now generally accepted that the Ankura thoracic stent graft was widely chosen in China when facing with economic issues.

In conclusion, satisfactory surgical results were obtained for this patient diagnosed as penetrating aorta ulcer associated with an ILVA using TEVAR remedy. This technique is an alternative to elephant trunk for PAU with an ILVA and no need for construction of bypass. Even the follow-up results were significantly acceptable while the period was relatively short, long-term follow-up is required to confirm the durability of this technique for PAU with an ILVA.

## Acknowledgments

We are very appreciated to the patient described in this case report and his family for providing informed consent and his cooperation.

## Author contributions

**Conceptualization:** Weijian Fan, Jianjie Rong.

**Data curation:** Weijian Fan, Chuanyong Li, Guangfeng Zheng.

**Formal analysis:** Weijian Fan, Chuanyong Li.

**Funding acquisition:** Weijian Fan, Chuanyong Li.

**Investigation:** Weijian Fan, Guangfeng Zheng.

**Methodology:** Guangfeng Zheng.

**Project administration:** Guangfeng Zheng.

**Resources:** Guangfeng Zheng.

**Software:** Guangfeng Zheng.

**Supervision:** Zhichang Pan.

**Validation:** Zhichang Pan.

**Visualization:** Zhichang Pan, Jianjie Rong.

**Writing – original draft:** Weijian Fan, Jianjie Rong.

**Writing – review & editing:** Weijian Fan, Jianjie Rong.
